# Muscle Mass and Gait Characteristics in Older Women Fallers vs. Non-Fallers

**DOI:** 10.3390/jcm10173924

**Published:** 2021-08-30

**Authors:** Yu-Ching Lin, Ing-Jy Tseng, Yi-Chien Lu, Shao-Wei Yang, Chia-Chi Wu, Yen-Nung Lin, Wing P. Chan

**Affiliations:** 1 Department of Medical Imaging and Intervention, Chang Gung Memorial Hospital, Keelung and Chang Gung University, Tayoun 33001, Taiwan; yuching1221@gmail.com; 2 School of Gerontology Health Management, College of Nursing, Taipei Medical University, Taipei 11031, Taiwan; ingjy@tmu.edu.tw; 3 Department of Radiology, Wan Fang Hospital, Taipei Medical University, Taipei 11696, Taiwan; 106263@w.tmu.edu.tw (Y.-C.L.); shaoweiyang0918@gmail.com (S.-W.Y.); 4 Department of Pathology, National Cheng Kung University Hospital, College of Medicine, National Cheng Kung University, Tainan 70403, Taiwan; chiachiwu0508@gmail.com; 5 Department of Physical Medicine and Rehabilitation, Wan-Fang Hospital, Taipei Medical University, Taipei 11696, Taiwan; solin@w.tmu.edu.tw; 6 Institute of Injury Prevention and Control, Taipei Medical University, Taipei 11031, Taiwan; 7 Department of Radiology, School of Medicine, College of Medicine, Taipei Medical University, Taipei 11696, Taiwan

**Keywords:** lean muscle mass, gait parameters, fall risk, older women

## Abstract

Background: Falling is a major public health concern of elderly people. We aimed to determine if lean mass and spatiotemporal gait parameters could predict the risk of falling in elderly women and also study the relationships between lean mass and gait characteristics. Methods: Twenty-four community women were prospectively recruited (mean age, 72.30 ± 5.31 years). Lean mass was measured using dual-energy fan-beam X-ray absorptiometry. Gait characteristics were assessed using spatiotemporal analysis. Fall risks were assessed using the Berg Balance Scale (BBS) and the Falls Efficacy Scale-International. Fall histories were recorded. Appropriate statistical analyses were applied to determine lean mass and gait characteristics in predicting the risk of fall and the associations between lean mass and gait characteristics. Results: There were 14 participants (58.33%) with fall histories. Patients with fall histories had a significantly narrower base of support and lower BBS score. However, only the base of support was significantly associated with fall risk (odds ratio, 0.415; *p* = 0.022). Lean mass was significantly negatively associated with proportion of swing phase and positively associated with proportions of stance and double-support phases. Conclusion: Fall risk among elderly women can be predicted using base of support, where a narrower base predicts a greater fall risk. Although the lean mass was not related to risk of fall, lean mass is still related to some gait characteristics.

## 1. Introduction

Fall-related fractures and injuries are among the major public health concerns of older people [1,2,3]. Reports show that on an annual basis, 60% of community-based older people fall, and 40% of those are recurrent [4,5,6]. The high prevalence of falls in the elderly may result in restricted mobility, prolonged hospitalization, anxiety, depression, and eventually impact on their quality of life [7]. Moreover, falls are the leading cause of traumatic brain injury and fracture in the elderly, and these injuries had a high mortality rate [8]. The European Union reported that falls represent 53% of all injury deaths in the elderly [9]. As the aging population expands worldwide, the healthcare costs of fall-related injuries increase [10,11,12], creating a desperate need for an accurate assessment tool for predicting falls among older people.

The three primary factors related to fall risk among elderly people are the intrinsic (patient-related), extrinsic (environment-related), and behavioral (activity-related) factors [13]. Intrinsic factors play major roles in both initial and recurrent falls, which often reduce mobility and physical activity in older people [14]. Restricting the activities of older people typically contributes to loss of muscle mass and muscle function, and it also alters gait patterns [14,15,16]. Altered gait patterns not only increase fall risk but can also predict the risk of falling [15,16]. Larger step length and greater walking speed are reportedly associated with increased fall risk [15]; therefore, those with fall histories tend to adopt more cautious walking patterns by decreasing step length and walking speed [16]. Notably, poor balance in elderly people also contributes to restricted activities and greater fall risk [17]. While increased fall risk is multifactorial and could be related to poor muscle mass, altered gait, and poor balance, studies of the relationships between those three factors remain limited. We hypothesize that muscle mass, gait characteristics, and poor balance will independently predict fall risk. Identifying the factors related to falls will allow early treatment intervention or customized specific treatment for the elderly. For example, if muscle loss increases fall risks, the elderly should focus on a nutrient supplement. While, if gait characteristic change increase fall risks, then the elderly should focus on gait rehabilitation. Thus, understanding the factors associated with falls is essential for the clinician to improve the quality of life in the elderly.

The aims of this study were to compare how lean mass, gait characteristics, balance, and fear of falling perform as predictors of fall risk in older women and to examine the relationships between lean mass and gait characteristics.

## 2. Materials and Methods

### 2.1. Study Design

We designed a prospective cross-sectional study to examine the following parameters: lean mass, gait characteristics, and fear of falling, with fall risk. We first divided participants into fallers and non-fallers, and measured parameters were compared between these two groups. Then, the measured parameters with a significant difference were further assessed for their value in predicting falls. Lastly, to understand the impact of gait on lean mass, we analyzed the relationship between gait and lean mass.

Lean muscle mass was measured using dual-energy fan-beam X-ray absorptiometry (DXA). Basic gait characteristics were acquired using spatiotemporal analysis. Balance was assessed using the Berg Balance Scale (BBS) [18], and fear of falling was assessed using the Falls Efficacy Scale-International (FES-I) [19,20]. Fall histories were obtained via patient recall.

### 2.2. Participants

This study was performed in accordance with the good clinical practice guidelines and the Declaration of Helsinki, and it was conducted after approval by the Taipei Medical University-Joint Institutional Review Board (Number: 201407017). All participants signed written informed consent.

We prospectively recruited 24 women from a single community, applying several inclusion criteria: Participants had no history of dementia (≤0.5 on the Clinical Dementia Rating scale) and adequate visual ability to read so that they could understand and consent to this study. Participants were able to walk more than 100 m without aids so that they could be able to perform spatiotemporal gait examination. Participants should live independently in the community so that they could come to the hospital to conduct study-related examinations. Exclusion criteria were history of hip or knee surgery, neurologic disease (e.g., Parkinson’s disease, stroke, multiple sclerosis), musculoskeletal disability, and cardiovascular disease (e.g., heart failure, coronary artery disease, arrhythmia).

### 2.3. Anthropometric Measures

Age, weight, height, and body mass index (BMI; weight in kilograms divided by the squared height in meters) were obtained. The first two were taken with the participant clad in light clothing and barefoot.

### 2.4. Total Body Composition

Total body composition was found using DXA equipment (Lunar Prodigy; GE Healthcare, Madison, WI, USA). Scan mode (standard, thin, or thick) was selected automatically by the scanner software based on body size and BMI. Scans were analyzed using enCORE Software, version 15 (GE Lunar). All measures were taken by experienced technicians certified by the International Society for Clinical Densitometry, and all protocols and procedures were strictly followed. All participants wore light indoor clothing and removed shoes and all other items that could interfere with DXA results. Each participant was positioned according to International Society for Clinical Densitometry guidelines [21].

Analysis of the DXA results yielded total lean mass (TLM; kg), total fat mass (TFM; kg), and total bone mass (TB; kg). Appendicular skeletal muscle (ASM; kg) was calculated by summing the bilateral arm lean mass (ALM; kg) with the bilateral leg lean mass (LLM; kg). Each was normalized by dividing by the squared height of the participant (m^2^), forming the respective mass indices (kg/m^2^) TLMI, TFMI, TBI, ASMI, ALMI, and LLMI. The precision error for this institute is lumbar spine < 1.9% and femoral neck < 2.5%.

### 2.5. Spatiotemporal Gait Parameters

Gait experiments were conducted in the gait lab at our institute. The GAITRite^®^ system (CIR Systems Inc., Clifton, NJ, USA) was employed to evaluate basic gait characteristics. We used the GAITRite^®^ system because it had been reported to have strong concurrent validity and test–retest reliability [22,23].

Spatiotemporal gait parameters (velocity, cadence, swing phase, stance phase, double-support phase, stride length, and base of support) were processed and stored using the application software. Across five trials, participants were directed to walk forward on the mat at their usual pace, utilizing both directions, by giving the instruction, “Please walk to the cone at your usual speed, go!” The mat was a 5.0 m GAITRite^®^ carpet on which the beginning and end of the course were delimited by cones placed 2 m from each end, demarking acceleration and deceleration zones. Data from all trials were combined per participant and considered a single test.

### 2.6. Berg Balance Scale

A valid and reliable objective measure of dynamic balance, the BBS is a 14-item test designed to measure the balance of older adults, assessing their performance of a specific functional task. Each item is scored from 1 to 4, allowing a maximum of 56 points. Fewer points indicate a greater danger to the individual’s gait stability.

### 2.7. Falls Efficacy Scale-International

Fear of falling was assessed using the validated Chinese version of the FES-I developed by Kwan et al. [19,20], a 16-item instrument developed by the Prevention of Falls Network Europe to augment the original 10-item Falls Efficacy Scale. It assesses an individual’s confidence (1, not concerned to 4, very concerned) in performing various basic or more complex/demanding physical and social daily living activities without falling.

### 2.8. Fall History

A fall was defined as unintentionally coming to the ground or floor from a standing height, except for conditions such as loss of consciousness, sudden onset of paralysis (stroke or epileptic seizure), or a violent blow. High-velocity falls were also not considered falls. In the initial interview, participants were asked, ‘‘During the past 12 months, have you fallen and landed on the floor or ground or fallen and hit an object like a table or stair while not doing sports in a standing height?’’

### 2.9. Statistical Analysis

The Mann–Whitney test was used to compare muscle mass, gait parameters, BBS, and FES-I between fallers and non-fallers, and a multivariate logistic regression model is used to assess their predictive values. Furthermore, Spearman’s correlation was used to evaluate associations between the body composition measures TLMI and TBI and the spatiotemporal gait characteristics BBS score and FES-I score. Statistical analysis was performed using SPSS 19.0 (SPSS Inc., Chicago, IL, USA) for Windows.

## 3. Results

Mean (standard deviation) age of the 24 participants was 72.30 years (range, 65 to 85 years). Fourteen participants (58.33%) were classified as fallers, and this group did not differ from non-fallers in age, BMI, postmenopausal time, TLMI, TFMI, ASMI, or FES-I. Fallers had a significantly narrower mean base of support (7.56 cm vs. 10.47 cm, *p* = 0.006) and a significantly lower mean BBS score (54.50 vs. 55.20, *p* = 0.039) compared to non-fallers (Table 1).

The multivariate analysis showed that both TFMI (odds ratio, 4.990 × 10^20^; 95% confidence interval, 1.592–1.564 × 10^41^; *p* = 0.045) and the base of support (odds ratio, 0.415; 95% confidence interval, 0.195–0.881; *p* = 0.022) were significantly associated with fall risk. No other spatiotemporal gait parameter was predictive of fall risk (Table 2).

Notably, TLMI and the other lean mass variables (ALMI, LLMI, and ASMI) were significantly associated with three spatiotemporal gait parameters: the swing phase (inverse relationship), stance phase (positive relationship), and double-support phase (positive relationship). In addition, TBI showed significant positive associations with gait velocity and stride length. Finally, BBS was associated with only TBI, and FES-I was not associated with any DXA measure (Table 3).

## 4. Discussion

Falls are common in the elderly; thus, this study investigates the relationship between muscle mass with gait, balance, and fall and assesses the performance of these factors in predicting the risk of falls in elderly women. Our results show that women with fall histories have a significantly narrower mean base of support and a significantly lower mean BBS score, but only the TFMI and base of support is predictive of fall risk. There is a trend of lean mass related to fall, but it did not reach a significant *p*-value; this may be associated with the small sample population. Moreover, lean mass is related to a more cautious gait, since the greater lean mass participants are associated with a shorter swing phase and longer stance and double-support phases. Finally, gait velocity is significantly positively associated with TBI, suggesting that better muscle performance positively impacts bone health.

Increased fall risk among older people is commonly associated with increased double-support time, decreased gait speed, increased stride length, and increased double-support time and base of support [24,25]. Double-support time increases when the time spent balancing on one leg decreases, and gait speed declines to allow more time to react to obstacles and environmental changes, which can cause a loss of balance. A shorter stride length minimizes the forward excursion of the center of mass beyond the base of support and promotes stability. A broader base of support recaptures the center of mass, which is important for older people, given that sideways falls occur during single-leg support [25].

This study shows that the base of support is one of the fall risk predictors among older women. This spatiotemporal gait parameter has been described as the horizontal stride width during the double-support phase (when both feet are in contact with the ground) [26]. It is commonly assumed that a broader base of support increases, rather than decreases, stability [27], and this study agrees in that a narrower base of support predicts a greater fall risk. Maki et al. [25] reported that changes in spatiotemporal gait parameters are more closely related to a pre-existing fear of falling than future falls; therefore, fear-related gait changes increase stability and reduce the likelihood of falling [25]. Based on fall histories and not the fear of falling, we hypothesized that in our cohort, the inappropriate gait characteristic of a narrow base of support could be the primary reason that this older population has poor balance and that it is the cause of their falls. It might be essential that older women adjust their gaits to include a broader base of support, thus improving gait stability and preventing future falls.

This study also shows that in older women, lean mass is positively associated with the proportions of the stance and double-support phases, implying that greater lean mass lengthens the stance and double-support phases. These gait adaptations are reportedly related to aging [28,29]. We observe that older women with greater lean mass are inclined to have more cautious gait, and we further propose that increasing lean mass alone might not be efficient in preventing fall or aging gait changes in older women; improvements in muscle function or power might also be necessary for improving gait stability.

Although the base of gait can predict falls, correcting the walk habitat may be challenging in the elderly. The selection of proper footwear may assist the elderly in achieving better gait stability [30,31]. Footwear plays an essential role in postural and dynamic instability by facilitating somatosensory feedback to the foot [30,31]. However, highly structured and supportive shoes may limit the tactile and proprioceptive system of the foot and affect the control of gait in the elderly [30,31]. The previous literature report that minimal shoes might be more beneficial for stability and physical function in the elderly than wearing conventional shoes [30,31].

Finally, the significant positive association between gait velocity and TBI suggests that elderly women with faster gaits will have greater bone mineral densities. This agrees with reports that gait speed might have an independent osteogenic effect associated with a greater whole-body bone density [30]. Greater bone density is related to musculoskeletal loading on the bone and might promote bone formation and attenuate bone loss [31] because osteoporosis is the primary cause of fractures in older people, possibly increasing the mortality rate and negatively affecting quality of life [30]. Although optimal bone health may be obtained from nutrition (such as vitamin D and calcium) and hormones supplement [32], this study also demonstrated that exercise (such as increased gait speed) could have a role in improving bone mineral content. However, in the osteoporotic elderly, increasing gait speed to improve bone mineral content must be applied with caution or even avoided, since these patients had fragile bones that were prone to fracture in low-velocity trauma.

This study has limitations. Fall histories could be obtained only through patient recall, which can lead to bias. Additionally, this study group included women only; the behaviors of men might yield different results. However, use of a single sex can result in more homogenous analyses. This prospective study did not obtain medication history for any of the participants. Thus, whether the elderly was under osteoporotic treatment was not known. Finally, the sufficient sample size to detect differences in the base of support between non-fallers and fallers, with a power of 80%, was 42. Meanwhile, our sample size was relatively small, which could lead to statical bias and overestimates in prediction values. The small sample size is related to recruitment difficulty. Most elderly were not willing to spend time on research questionnaires and examinations.

## 5. Conclusions

Among elderly women, fallers have narrower bases of support and lower BBS scores compared to non-fallers, but TFMI and base of support is a predictor of fall risk. Lean mass is unrelated to fall risk and related to more cautious gait.

## Figures and Tables

**Table 1:** Differences in mean (SD) lean mass and spatiotemporal gait parameters of fallers vs. non-fallers.

Characteristic	All Participants (*n* = 24)	Non-Fallers (*n* = 10)	Fallers (*n* = 14)	*p* Value
Age (year)	72.30 (5.31)	73.26 (6.29)	71.62 (4.61)	0.520
Height (cm)	151.40 (5.43)	151.85 (6.94)	151.09 (4.30)	0.500
Weight (kg)	55.42 (8.58)	55.31 (10.15)	55.50 (7.67)	0.930
Body mass index (kg/m^2^)	24.11 (3.16)	23.82 (2.92)	24.33 (3.41)	0.815
Postmenopausal time (year)	48.21 (5.27)	48.70 (2.00)	47.86 (6.78)	0.244
Total body composition				
Total lean mass index	14.26 (1.07)	14.22 (1.22)	14.28 (0.99)	0.815
Total fat mass index	9.21 (2.30)	8.84 (2.05)	9.49 (2.51)	0.709
Total bone mineral index	0.74 (0.09)	0.76 (0.10)	0.72 (0.07)	0.341
ASMI	6.00 (0.66)	5.85 (0.66)	6.10 (0.66)	0.349
Spatiotemporal gait parameters				
Velocity (cm/s)	299.27 (64.00)	293.96 (65.84)	303.07 (64.88)	0.953
Cadence (steps/min)	331.34 (31.17)	330.43 (32.83)	331.98 (31.18)	0.815
Swing phase (%)	37.59 (1.61)	37.64 (1.43)	37.55 (1.77)	0.725
Stance phase (%)	62.54 (1.74)	62.31 (1.35)	62.71 (2.01)	0.500
Double-support phase (%)	25.03 (3.21)	24.59 (2.98)	25.35 (3.43)	0.520
Stride length (cm)	107.93 (16.51)	105.67 (17.88)	109.55 (15.95)	0.558
Base of support (cm)	8.77 (2.45)	10.47 (2.19)	7.56 (1.87)	0.006 *
Balance				
Berg Balance Scale score	54.79 (1.74)	55.20 (1.87)	54.50 (1.65)	0.039 *
Fall Risk				
FES-I score	38.18 (10.87)	36.78 (13.42)	39.15 (9.17)	0.815

FES-I = Falls Efficacy Scale-International, ASMI = appendicular skeletal muscle index. * *p* < 0.05. Statistical analysis was performed with Mann–Whitney test.

**Table 2:** Regression analysis and odds ratio (OR) of each body composition and gait parameter.

	OR (95% CI)	*p*-Value
Total body composition		
Total lean mass index	0.000 (0.000–62.064)	0.071
Total fat mass index	4.990 × 10^20^ (1.592–1.564 × 10^41^)	0.048 *
Total bone mineral index	8.183 × 10^134^ (-)	0.362
Spatiotemporal gait parameters		
Velocity	1.002 (0.989–1.016)	0.726
Cadence	1.002 (0.975–1.029)	0.902
Swing phase	0.963 (0.575–1.613)	0.885
Double-support phase	1.081 (0.831–1.406)	0.561
Stride length	1.015 (0.965–1.068)	0.564
Base of support	0.415 (0.195–0.881)	0.022 *
Balance		
Berg Balance Scale score	0.753 (0.417–1.360)	0.347
Fall Risk		
FES-I score	1.021 (0.942–1.107)	0.608

CI = confidence interval; FES-I = Falls Efficacy Scale-International. * *p* < 0.05. Statistical analysis was performed with multivariate logistic regression.

**Table 3:** Correlation between muscle volume and spatiotemporal gait parameters.

Variable	TLMI	ALMI	LLMI	ASMI	TBI
Spatiotemporal gait parameters					
Velocity (cm/s)	−0.116	−0.290	−0.044	−0.102	0.456 *
Cadence (steps/min)	−0.260	−0.390	−0.323	−0.366	0.237
Swing phase	−0.524 **	−0.414 *	−0.536 **	−0.493 *	0.085
Stance phase	0.595 **	0.571 **	0.607 **	0.599 **	0.014
Double-support phase	0.588 **	0.519 **	0.549 **	0.530 **	−0.074
Stride length	−0.094	−0.206	0.032	0.002	0.465 *
Base of support	0.295	−0.061	0.163	0.092	0.422
Balance					
BBS	−0.030	0.244	0.195	0.195	0.463 *
Fall Risk					
FES-I	0.299	0.181	0.142	0.158	0.179

TLMI = total lean mass index; ALMI = arm lean mass index; LLMI = leg lean mass index; ASMI = appendicular skeletal muscle index; TBI = total bone mass index; BBS = Berg Balance Scale; FES-I = Falls Efficacy Scale-International. * *p* < 0.05; ** *p* < 0.01. Statistical analysis was performed with Spearman’s Correlation.

## Data Availability

The data presented in this study are available on request from the corresponding author.
